# Study protocol for a multicentre prospective cohort study to identify predictors of adverse outcome in older medical emergency department patients (the Risk Stratification in the Emergency Department in Acutely Ill Older Patients (RISE UP) study)

**DOI:** 10.1186/s12877-019-1078-2

**Published:** 2019-03-04

**Authors:** Noortje Zelis, Jacqueline Buijs, Peter W. de Leeuw, Sander M. J. van Kuijk, Patricia M. Stassen

**Affiliations:** 1 Department of Internal Medicine and Gastroenterology, Zuyderland Medical Centre, Heerlen, the Netherlands; 2 Department of Internal Medicine, Division of General Internal Medicine, Section Acute Medicine, Maastricht University Medical Centre, Maastricht University, Maastricht, the Netherlands; 3 School of CAPHRI, Maastricht University Medical Centre, Maastricht University, Maastricht, the Netherlands; 4 CARIM School for Cardiovascular Diseases, Maastricht University Medical Centre, Maastricht University, Maastricht, the Netherlands; 5 Department of Clinical Epidemiology and Medical Technology Assessment, Maastricht University Medical Centre, Maastricht University, Maastricht, the Netherlands; 6 Department of Internal Medicine, K&E1, Zuyderland Medical Centre, Heerlen, Postbus 5500, 6130 MB Sittard-Geleen the Netherlands

**Keywords:** Risk stratification, Older patients, Emergency department, Prognosis

## Abstract

**Background:**

Older patients (≥65 years old) experience high rates of adverse outcomes after an emergency department (ED) visit. Reliable tools to predict adverse outcomes in this population are lacking. This manuscript comprises a study protocol for the Risk Stratification in the Emergency Department in Acutely Ill Older Patients (RISE UP) study that aims to identify predictors of adverse outcome (including triage- and risk stratification scores) and intends to design a feasible prediction model for older patients that can be used in the ED.

**Methods:**

The RISE UP study is a prospective observational multicentre cohort study in older (≥65 years of age) ED patients treated by internists or gastroenterologists in Zuyderland Medical Centre and Maastricht University Medical Centre+ in the Netherlands.

After obtaining informed consent, patients characteristics, vital signs, functional status and routine laboratory tests will be retrieved. In addition, disease perception questionnaires will be filled out by patients or their caregivers and clinical impression questionnaires by nurses and physicians. Moreover, both arterial and venous blood samples will be taken in order to determine additional biomarkers. The discriminatory value of triage- and risk stratification scores, clinical impression scores and laboratory tests will be evaluated.

Univariable logistic regression will be used to identify predictors of adverse outcomes. With these data we intend to develop a clinical prediction model for 30-day mortality using multivariable logistic regression. This model will be validated in an external cohort.

Our primary endpoint is 30-day all-cause mortality. The secondary (composite) endpoint consist of 30-day mortality, length of hospital stay, admission to intensive- or medium care units, readmission and loss of independent living.

Patients will be followed up for at least 30 days and, if possible, for one year.

**Discussion:**

In this study, we will retrieve a broad range of data concerning adverse outcomes in older patients visiting the ED with medical problems. We intend to develop a clinical tool for identification of older patients at risk of adverse outcomes that is feasible for use in the ED, in order to improve clinical decision making and medical care.

**Trial registration:**

Retrospectively registered on clinicaltrials.gov (NCT02946398; 9/20/2016).

**Electronic supplementary material:**

The online version of this article (10.1186/s12877-019-1078-2) contains supplementary material, which is available to authorized users.

## Background

Older patients (≥65 years of age) constitute an increasing population in emergency departments (EDs). They experience more adverse outcomes than younger ED patients [1–3] as their ED visits are often highly urgent and followed by hospitalization, Intensive Care Unit (ICU) admission, readmission, and mortality (up to 10% within 3 months). In older ED patients with internal medicine (medical) problems, mortality is even higher (23.8% in 3 months) [4].

Risk stratification can be used to identify ED patients who are at high risk of adverse outcomes. This may lead to improvement in medical care and outcome by early start of interventions [5]. Several risk stratification scores have been developed for the older ED population, such as the Identification of Seniors at Risk (ISAR) [6] or Triage Risk Stratification Tool (TRST) [7]. Unfortunately, these scores do not accurately identify those who experience adverse outcomes (areas under the curve (AUCs) range from 0.59–0.74) [8–10]. Triage systems (e.g. Manchester Triage System (MTS) [11]) are also used in the ED population but they tend to undertriage older patients [12]. Furthermore, risk stratification scores either applicable to the general ED population (e.g. Acute Physiology and Chronic Health Evaluation II (APACHE II) score [13]) or to patients with specific diseases (e.g. Abbreviated Mortality ED Sepsis (abbMEDS) score [14] for sepsis) are used. However, these are not validated in the older ED population.

It may be possible that older patients at risk of adverse outcomes can be identified by assessing the disease perception of patients (or caregivers) or clinical intuition or impression of physicians and/or nurses. Indeed, both disease perception and clinical impression are shown to be associated with mortality and morbidity [15–20]. Unfortunately, most studies were conducted in other clinical settings than the ED (e.g. admission units and ICUs) [4, 17, 20] and in younger patients [17, 18, 20]. A second method to assess clinical impression is to ask the ‘surprise question’ (SQ): ‘Would I be surprised if this patient died within the next 12 months?’. The SQ has been studied in cancer and renal failure patients but its diagnostic accuracy for one-year mortality varies considerably [21]. The predictive value of the SQ for short-term mortality in older medical ED patients is unknown.

Laboratory tests may also be useful for identification of older patients at risk of adverse outcomes [4, 22]. In addition, non-routine laboratory tests, such as lactate, high-sensitivity cardiac troponin T (hs-cTnT), N-terminal pro-B-type natriuretic peptide (NT-pro-BNP), procalcitonin (PCT) and d-dimer, may be valuable predictors of adverse outcome as well [23–27]. Since these tests are indicators of serious conditions and diseases (e.g. tissue hypoperfusion, myocardial injury, bacterial infection and thromboembolism) and often present in the older ED population, we hypothesize that these tests can be useful as predictors. Until now, most studies regarding the predictive value of these tests were performed retrospectively [24, 28–33] or in selected, mostly younger, patients [30–38].

We hypothesize that in the early stage of an ED visit, when important treatment decisions have to be made, several factors can predict adverse outcomes. The aims of this multicentre, prospective study are to 1) identify early predictors of adverse outcome in older ED patients, and 2) develop a clinical prediction model for 30-day mortality.

## Methods/design

### Study design and setting

This prospective multicentre observational cohort study will take place at the EDs of Zuyderland Medical Centre (MC) Heerlen and Maastricht University Medical Centre+ (MUMC+), in the Netherlands. Zuyderland MC is a secondary teaching hospital with 635 beds and 30,000 ED visits/year. MUMC+ is a secondary and tertiary teaching hospital with 700 beds and approximately 23,000 ED visits/year.

### Study population

All patients ≥65 years of age, assessed and treated by an internist or gastroenterologist at the EDs during the study period, are eligible for study inclusion. We chose medical ED patients because these patients are at high risk of adverse outcomes after an ED visit [4]. We intend to include 450 patients in Zuyderland MC starting from July 2016, and 150 patients in MUMC+ from September 2016.

Inclusion criteria:

- Age ≥ 65 years
- Treatment by an internist, gastroenterologist (or emergency physician under supervision of an internist/gastroenterologist)
- Informed consent

Exclusion criteria:

- Earlier participation in this study
- No informed consent
- Inability to speak Dutch or English
- Admission to a ward of another specialty than internal medicine/gastroenterology

In case a patient is unable to provide informed consent, e.g. in case of delirium, dementia or when a patient is too severely ill to answer the questions, a legal representative can provide informed consent. A legal representative can either be a legal guardian or an immediate family member including their spouse, adult children, parents or adult siblings. The determination, whether or not a patient can provide informed consent for them self, will be based on expect opinion by the attending physician or investigator. Objection by an incapacitated patient or his/her representatives will always lead to exclusion from the study and analysis.

### Objectives and outcome

#### Objectives

1. Evaluation of the discriminatory value for adverse outcomes of:
  - triage and risk stratification scores:
    Triage score: MTS [11]
    General risk stratification scores: APACHE II [13], ISAR score [6] and ISAR-Hospitalised Patients (ISAR-HP) score [39]
    Disease specific scores: Glasgow-Blatchford Bleeding Score (GBS) [40], abbMEDS score [14], Sepsis-related Organ Failure Assessment (SOFA) score [41] and Confusion, Urea, Respiration, Blood pressure, Age > 65 years (CURB-65) score [42]
  - clinical impressions of nurses and physicians, SQ and disease perception of patients
  - routine and non-routine laboratory results
2. Development and validation of a prediction model for short-term mortality and test the predictive ability of this model for the other adverse outcomes/endpoints

#### Outcomes

Primary endpoint:

- 30-day (both in- and out-hospital) all-cause mortality

Secondary endpoints:

- Secondary composite endpoint:
  30-day mortality
  Length of hospital stay (LOS) > 7 days
  Intensive or medium care unit (ICU/MCU) admission
  Unplanned readmission within 30 days after discharge
  Loss of independent living (e.g. discharge to a nursing home/hospice or with palliative care in previously community dwelling patients)
- 1-year all-cause mortality

### Study procedures

#### Inclusion of patients

**Fig. 1:**
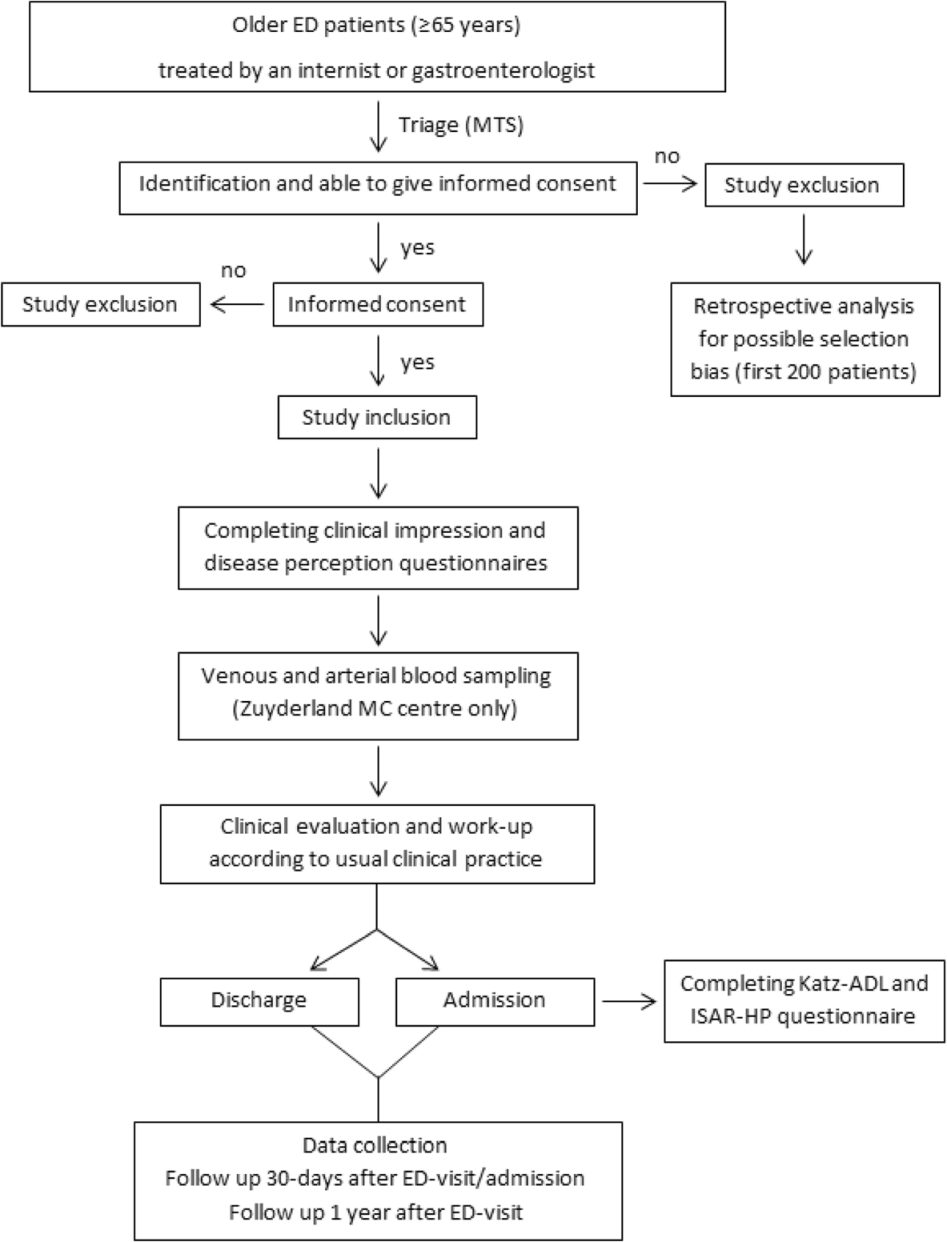
Flow chart of study procedure

After arrival at the ED, all eligible patients will receive an information brochure and will be asked to participate in the study by the attending physician or investigator. Informed consent must be signed by the patient or his/her legal representative before entering the study. Figure 1 details the study procedure.

#### Questionnaires

The patient/caregiver will receive a questionnaire at the ED that should be filled out as soon as possible. This questionnaire (Additional file 1) contains four questions regarding disease and health perception. The nurse and attending physician both receive a similar questionnaire (Additional files 2 and 3) that has to be filled out before history taking and physical examination and without knowing any diagnostic results. This questionnaire contains six questions regarding the first clinical impression including the SQ. When a patient is admitted to the hospital, a fourth questionnaire (Additional file 4), containing ten questions about the patients’ daily functioning, will be filled out the next day. This questionnaire is used to calculate the ISAR-HP and Katz Activities of Daily Living (ADL) [43] index score. The results of the questionnaires will only be available for the investigators.

#### Blood sample collection

In addition to routine blood sampling, two venous blood samples and an arterial blood gas sample will be collected at the ED. Venous blood samples will be stored in a freezer at − 20 degrees Celsius and will be analysed for hs-cTnT, NT-pro-BNP, PCT and d-dimer after 4–12 weeks. Results will be blinded for the physician. Results of the arterial blood gas and lactate level analysis will be presented to the attending physician. Additional venous and arterial blood sample collections will only be performed in Zuyderland MC.

#### Data collection and follow up

Study parameters will be retrieved from the patient’s medical electronic record and questionnaires. All patients will be followed up for 1 year to obtain long-term outcomes. The following parameters will be collected:

*Study parameters collected at the ED:*

- Demographics (age, sex)
- Date and time of ED visit and transport to the ED
- Comorbidities: Charlson Comorbidity Index [44], smoking status and presence of cardiovascular disease in family history
- Vital signs: heart rate, blood pressure, respiratory rate, oxygen saturation, temperature, Glasgow Coma Scale [45]
- First clinical impression (including the SQ) of the physician/nurse and disease perception of the patient/caregiver using questionnaires
- Cognitive functioning (dementia, mild cognitive impairment, delirium or normal) based on the diagnosis of a geriatrician and/or on medical records
- Number of visits to the hospital in the preceding year
- Medication use before the ED visit
- Time spent at the ED and the number of physician consultations and radiological examinations during ED stay
- Routine laboratory tests: glucose, creatinine, blood urea nitrogen, sodium, potassium, chloride, bicarbonate, calcium, phosphate, bilirubin, alkaline phosphatase, gamma- glutamyltransferase, aspartate transaminase, alanine transaminase, lactate dehydrogenase, albumin, c-reactive protein, hemoglobin, hematocrit, leukocyte count, platelet count, international normalized ratio and activated partial thromboplastin time
- Non-routine laboratory tests: arterial blood gas, lactate, hs-cTnT, NT-pro-BNP, PCT and d-dimer
- Triage score: MTS
- General risk stratification scores:
  1. APACHE II score
  2. ISAR score
- Disease specific stratification scores:
  1. GBS for upper gastrointestinal bleeding
  2. abbMEDS score for sepsis
  3. SOFA score for sepsis
  4. CURB-65 score for pneumonia

These scores were only calculated when the specific disease for which the score was developed was present.

- Diagnosis at the time of discharge from the ED

*Study parameters collected in admitted patients only:*

- Functional capability:
  1. Katz ADL index score
  2. ISAR-HP score
- Diagnosis at time of discharge from the hospital
- LOS (days)
- Living arrangement after discharge: e.g. community dwelling, nursing- or care home etc.

Follow up study parameters collected in all participants:

- ICU/MCU admission
- All-cause mortality within 30 days of the ED visit
- Readmission within 30 days after discharge
- Any new relevant medical condition within 1 year after ED visit (e.g. new diagnose of venous thromboembolism, cardiovascular disease or malignancy)

#### Possible selection bias

Because physicians must give priority to providing emergency care when the ED is crowded, we expect that not all possible candidates can be included. To investigate possible selection bias, we will retrospectively form a sample of non-included patients and collect the same data, except for the non-routine tests and questionnaires, as in our prospective cohort population. For practical reasons, we intend to include the first 200 non-included candidates in this retrospective sample. Patients who refused to participate in the study will not be included. Baseline characteristics (age and sex) will be analysed for all (non-included) candidates to investigate possible selection bias as well.

### Study analysis

#### Statistical analysis

First, patients characteristics and outcomes will be described. Continuous variables will be reported as means with standard deviations or medians with interquartile range and categorical variables as proportions. Valid percentages will be used when values are missing.

Secondly, we will quantify the ability of the risk-stratification scores, clinical impression scores and non-routine laboratory tests to discriminate between the presence and absence of the different endpoints separately using the area under the receiver operator characteristics curve (AUC-ROC). We will determine their diagnostic accuracy using sensitivity, specificity, positive and negative predictive values (PPV, NPV), likelihood ratios and Youden’s index. For the discriminatory value of risk-stratification scores and clinical impression scores, combined data from both centres will be used. For the non-routine laboratory tests, only data from patients recruited in Zuyderland MC will be used.

Third, we will identify possible predictors of adverse outcome using univariable logistic regression analyses. Odds ratios (OR) with 95% confidence intervals (95% CI) will be calculated. Continuous variables will be checked for nonlinearity and collinearity. In case of missing items, we will use stochastic regression imputation to impute these items using predictive mean matching.

#### Model development

We will develop a clinical prediction model for 30-day mortality using multivariable logistic regression with predictors that are deemed feasible. We consider predictors feasible when a parameter is available in at least 90% of the participants at the ED, reproducible and easily retrieved. Participants that are prospectively included in Zuyderland MC and MUMC+ will form the derivation cohort and their data will be used for model development. For external validation, we will retrospectively collect data of ED patients to form a validation cohort. For the development and validation of our model the Stiell criteria will be applied [46].

After external validation, we will test the predictive ability of the model for the secondary composite endpoint and test whether addition of clinical impression scores and/or non-routine laboratory tests results in a better prediction of adverse outcomes.

All data will be analysed using IBM SPSS Statistics for Windows, Version 24.0 (IBM Corp., Armonk, N.Y., USA) and R version 3.3.3.

#### Sample size calculation

Since we will be using logistic regression analysis to identify predictors of 30-day mortality, a minimum of 10 events per candidate predictor is suggested [47]. Expected 30-day mortality in older medical ED patients is assumed to be at least 10% based on previous studies in the Netherlands [4, 48]. Therefore, we decided to include 450 patients in Zuyderland MC and 150 patients in MUMC+ to form the derivation cohort. For external validation of our prediction model, we need approximately 100 events, and therefore, the sample size of the validation cohort will be based on the mortality rate of the derivation cohort (estimation: 800 patients).

### Trial status

As of 22/12/2018, the study is still ongoing; we are about to finish the inclusion of patients for the validation cohort and are completing 1 year follow up. A total of 603 participants are included in the derivation cohort from July 2016 until the beginning of February 2017 in the two participating centres. In Zuyderland MC, 450 patients, and in MUMC+, 153 patients are included.

## Discussion

Early risk stratification at the ED is extremely important to optimise treatment and improve outcomes in acutely ill older patients. To identify older patients with increased risk of adverse outcomes in an early stage, we need accurate predictors.

In the past decades several studies identified risk factors and predictors of adverse outcomes in the older ED population [4, 8, 49]. However, most of these studies were conducted in an unselected population of older patients, leading to conflicting results. For our study we chose medical ED patients because we assume that this group of patients represent a large group of patients who are highly at risk of adverse outcomes. Furthermore, we chose to evaluate more endpoints (including composite endpoints) because some adverse outcomes exclude others (i.e. dying in-hospital will prevent readmission). We are convinced that for quantification of the risk of adverse outcomes in the older population, more than one endpoint is needed for a reliable clinical appraisal. We consider patients older than 65 years to be old and we chose this cut off based on other well-known screening instruments such as the ISAR [6], the ISAR-HP [39] and TRST [7].

Development and validation of our prediction model for 30-day mortality will be according to the Stiell criteria [46]. Since reliable tools to predict short-term mortality are lacking there is need for such a prediction model (criterion 1). Before conduct of this study, internists, gastroenterologists and geriatricians were consulted on the need of a clinical tool, preferred outcomes to be studied and potentially meaningful predictors. The model will be derived according to methodologic standards and is intended to be easily implemented in routine ED care (criterion 2). Most of the predictors we will select will resemble the morbid state rather that the premorbid state because we hypothesize that that the severity of the disease for which the patient visits the ED (morbid state) may be more important than the premorbid state for prediction of short-term mortality. We aim to externally validate our prediction model in a different ED population (criterion 3). Once we have succeeded in developing and externally validating an accurate model, we intend to implement it into clinical practice by offering an online calculator, which may also be incorporated into a electronical file management system (criterion 4).

The main strengths of the RISE UP study are, in our opinion, its prospective multicentre study design in an ED setting and the use of composite endpoints. We aim to identify predictors of adverse outcomes in an early stage of presentation, in the ED setting, when important decisions need to be made. We will not only evaluate the predictive value of the clinical impression of both the patient, nurse and physician, but also that of routine and non-routine laboratory tests. A possible limitation is that we expect that we cannot include all potential candidates, due to crowding of patients at the ED or other logistic problems. Therefore, we will perform an additional analysis to investigate possible selection bias. In addition, this study will include internal medicine and gastroenterology patients only. If this study yields an accurate model, that model will have to be tested in the overall older ED population. Furthermore, we intend to implement the model into clinical practice by use of an online calculator. Possibly, this may also be incorporated into an electronical file management system or a mobile App.

In summary, the RISE UP study is a prospective multicentre cohort study that aims to identify predictors of adverse outcomes in older medical ED patients. The goal of this study is to develop a practical, feasible tool to identify older ED patients with an increased risk of adverse outcomes in an early stage, in order to improve their care in the future.

## Additional files

Additional file 1: Emergency department questionnaire for the patient or caregiver. Details the questionnaire of the patient/caregiver which should be filled out in the ED. This questionnaire contains questions regarding disease and health perception. (DOCX 50 kb)

Additional file 2: Emergency department questionnaire for the nurse. Details the questionnaire of the nurse which should be filled out in the ED before history taking and physical examination and without knowledge of the diagnostic results. This questionnaire contains questions regarding the first clinical impression including the surprise question. (DOCX 60 kb)

Additional file 3: Emergency department questionnaire for the physician. Details the questionnaire of the physician which should be filled out in the ED before history taking and physical examination and without knowledge of the diagnostic results. This questionnaire contains questions regarding the first clinical impression including the surprise question. (DOCX 62 kb)

Additional file 4: Functional capability assessment questionnaire. Details the questionnaire regarding the patient’s functional capability two weeks before admission. This questionnaire should be filled out during hospital stay and will be used to calculate the Katz Activities of Daily Living and Identification of Seniors at Risk - Hospitalised Patients score. (DOCX 60 kb)

## Abbreviations

abbMEDS: Abbreviated Mortality ED Sepsis
ADL: Activities of Daily Living
APACHE II: Acute physiology and chronic health evaluation II
AUC: Area under the curve
AUC-ROC: Area under the receiver operator characteristics curve
CI: Confidence intervals
CURB-65: Confusion, Urea, Respiration, Blood pressure, Age > 65
ED: Emergency Department
GBS: Glasgow-blatchford bleeding score
Hs-cTnT: High-sensitivity cardiac troponin T
ICU: Intensive care unit
ISAR: Identification of seniors at risk
ISAR-HP: Identification of seniors at risk - hospitalised patients
LOS: Length of hospital stay
MC: Medical Centre
MCU: Medium care unit
MTS: Manchester triage score
MUMC+: Maastricht University Medical Centre+
NPV: Negative predictive value
NT-pro-BNP: N-terminal pro-B-type natriuretic peptide
OR: Odds ratio
PCT: Procalcitonin
PPV: Positive predictive value
RISE UP: Risk Stratification in the Emergency Department in Acutely Ill Older Patients
SOFA: Sepsis-related organ failure assessment
SQ: Surprise question
TRST: Triage Risk Stratification Tool
