# Supplementary figures and images for: Study protocol for a multicentre prospective cohort study to identify predictors of adverse outcome in older medical emergency department patients (the Risk Stratification in the Emergency Department in Acutely Ill Older Patients (RISE UP) study)
Source: BMC Geriatr. 2019 Mar 4;19:65. doi: 10.1186/s12877-019-1078-2 (PMC6399878; doi:10.1186/s12877-019-1078-2)

**Additional file 1: Emergency department questionnaire for the patient or caregiver**

**
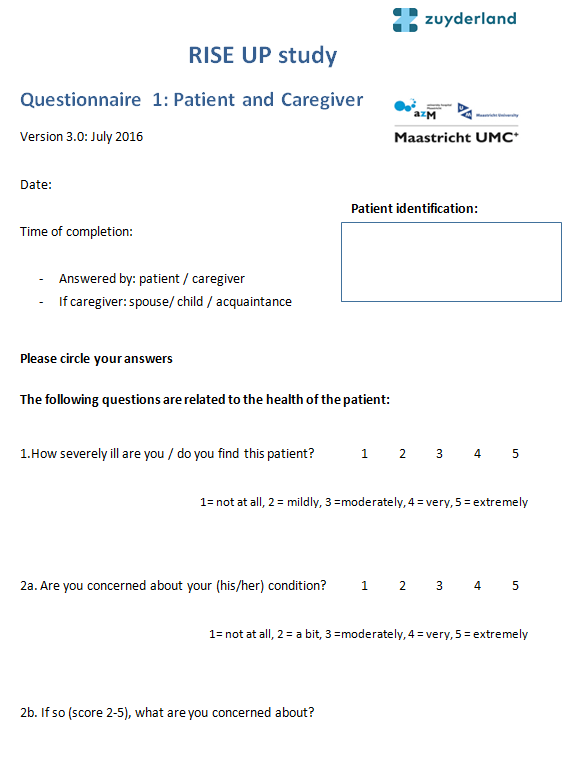
**

**
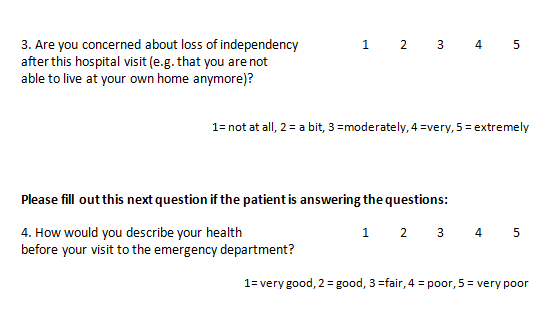
**

Supplement: Supplementary file 1 — Emergency department questionnaire for the patient or caregiver. Details the questionnaire of the patient/caregiver which should be filled out in the ED. This questionnaire contains questions regarding disease and health perception. (DOCX 50 kb) [file 12877_2019_1078_MOESM1_ESM.docx]

**Additional file 2: Emergency department questionnaire for the nurse**

**
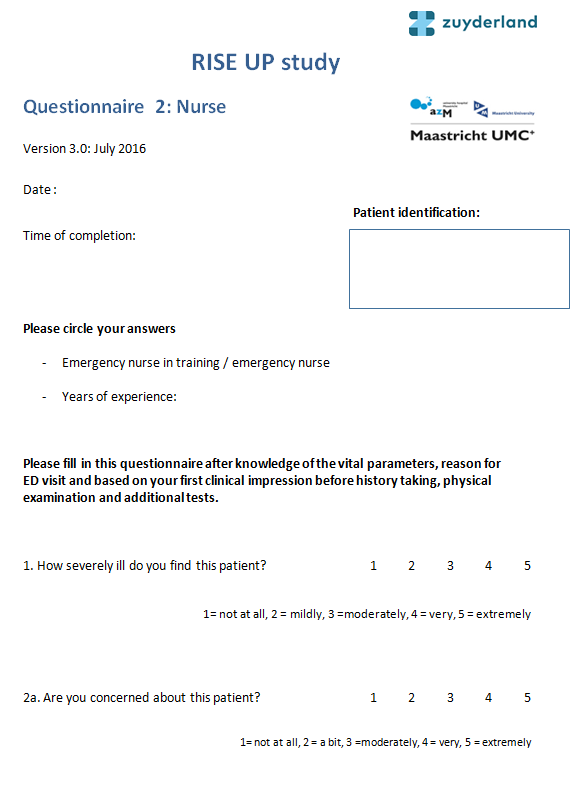
**

**
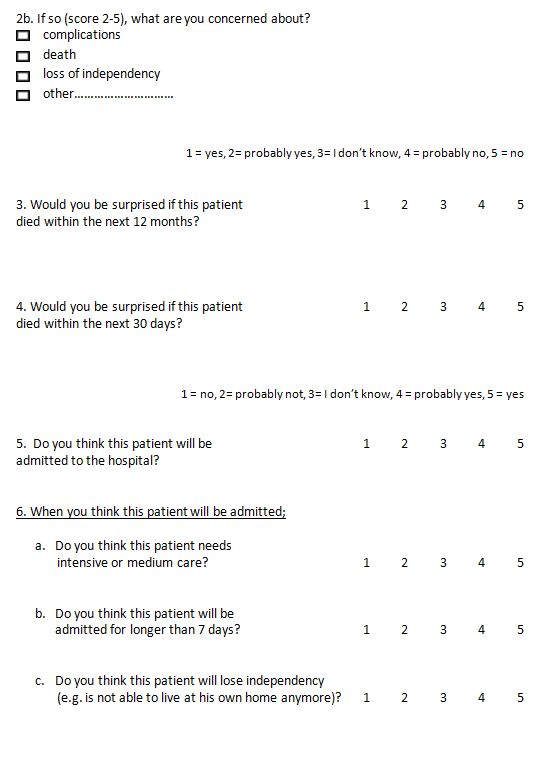
**

Supplement: Supplementary file 2 — Emergency department questionnaire for the nurse. Details the questionnaire of the nurse which should be filled out in the ED before history taking and physical examination and without knowledge of the diagnostic results. This questionnaire contains questions regarding the first clinical impression including the surprise question. (DOCX 60 kb) [file 12877_2019_1078_MOESM2_ESM.docx]

**Additional file 3: Emergency department questionnaire for the physician**

**
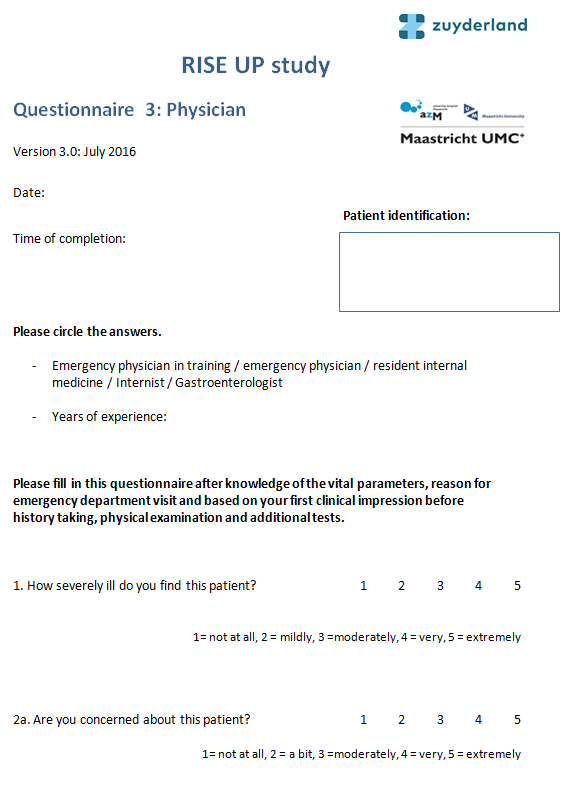
**

**
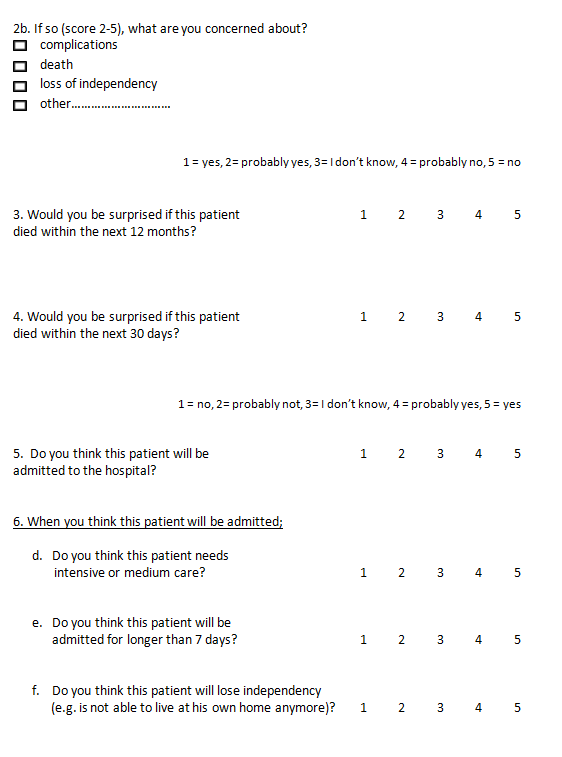
**

Supplement: Supplementary file 3 — Emergency department questionnaire for the physician. Details the questionnaire of the physician which should be filled out in the ED before history taking and physical examination and without knowledge of the diagnostic results. This questionnaire contains questions regarding the first clinical impression including the surprise question. (DOCX 62 kb) [file 12877_2019_1078_MOESM3_ESM.docx]
