# Supplementary material for: Study protocol for a multicentre prospective cohort study to identify predictors of adverse outcome in older medical emergency department patients (the Risk Stratification in the Emergency Department in Acutely Ill Older Patients (RISE UP) study)
Source: BMC Geriatr. 2019 Mar 4;19:65. doi: 10.1186/s12877-019-1078-2 (PMC6399878; doi:10.1186/s12877-019-1078-2)
Supplement: Supplementary file 4 — Functional capability assessment questionnaire. Details the questionnaire regarding the patient’s functional capability two weeks before admission. This questionnaire should be filled out during hospital stay and will be used to calculate the Katz Activities of Daily Living and Identification of Seniors at Risk - Hospitalised Patients score. (DOCX 60 kb) [file 12877_2019_1078_MOESM4_ESM.docx]

**Additional file 4: Functional capability assessment questionnaire**

**
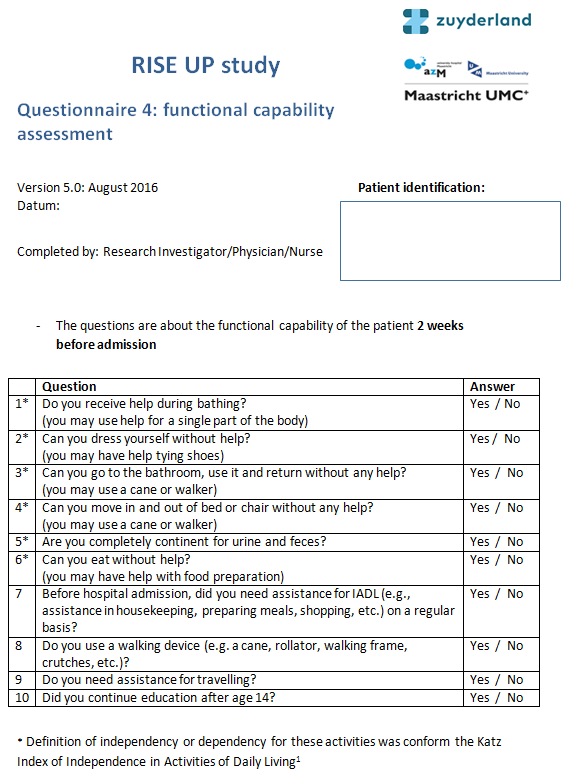
^[[1]](#footnote-1)^**

1. Katz S et. al., *Jama,* 1963 [↑](#footnote-ref-1)
